# Tightly-Coupled Integration of Multi-GNSS Single-Frequency RTK and MEMS-IMU for Enhanced Positioning Performance

**DOI:** 10.3390/s17112462

**Published:** 2017-10-27

**Authors:** Tuan Li, Hongping Zhang, Xiaoji Niu, Zhouzheng Gao

**Affiliations:** 1 GNSS Research Center, Wuhan University, 129 Luoyu Road, Wuhan 430079, China; tuanli@whu.edu.cn (T.L.); xjniu@whu.edu.cn (X.N.); 2 School of Land Science and Technology, China University of Geosciences, 29 Xueyuan Road, Beijing 100083, China; zhouzhenggao@whu.edu.cn; 3 German Research Centre for Geosciences (GFZ), Telegrafenberg, 14473 Postsdam, Germany

**Keywords:** multi-GNSS (GPS/BDS/GLONASS), single-frequency real-time kinematic (SF-RTK), MEMS-IMU, tight-coupled integration, integer ambiguity resolution

## Abstract

Dual-frequency Global Positioning System (GPS) Real-time Kinematics (RTK) has been proven in the past few years to be a reliable and efficient technique to obtain high accuracy positioning. However, there are still challenges for GPS single-frequency RTK, such as low reliability and ambiguity resolution (AR) success rate, especially in kinematic environments. Recently, multi-Global Navigation Satellite System (multi-GNSS) has been applied to enhance the RTK performance in terms of availability and reliability of AR. In order to further enhance the multi-GNSS single-frequency RTK performance in terms of reliability, continuity and accuracy, a low-cost micro-electro-mechanical system (MEMS) inertial measurement unit (IMU) is adopted in this contribution. We tightly integrate the single-frequency GPS/BeiDou/GLONASS and MEMS-IMU through the extended Kalman filter (EKF), which directly fuses the ambiguity-fixed double-differenced (DD) carrier phase observables and IMU data. A field vehicular test was carried out to evaluate the impacts of the multi-GNSS and IMU on the AR and positioning performance in different system configurations. Test results indicate that the empirical success rate of single-epoch AR for the tightly-coupled single-frequency multi-GNSS RTK/INS integration is over 99% even at an elevation cut-off angle of 40°, and the corresponding position time series is much more stable in comparison with the GPS solution. Besides, GNSS outage simulations show that continuous positioning with certain accuracy is possible due to the INS bridging capability when GNSS positioning is not available.

## 1. Introduction

The dual-frequency GPS real-time kinematic (RTK) technique is known to be used in many applications that require high accuracy positioning such as land surveying, precise agriculture and vehicular navigation. The carrier phase integer ambiguity resolution (AR) is a prerequisite to obtain centimeter-level high-precision positioning. Researches have shown that the dual-frequency GPS RTK can achieve rapid AR for short baselines in open-sky condition [1,2]. By contrast, the single-frequency RTK has relatively low ambiguity fixing rate and reliability due to the residual atmospheric error, multipath error and observation noise, especially in dynamic conditions [3]. However, the high cost of dual-frequency receivers greatly restrict potential applications.

With the rapid deployment of multiple GNSS constellations [4], RTK performance is expected to be significantly improved in terms of the availability, accuracy, reliability and continuity. The benefits from multiple GNSS constellations are obvious for RTK positioning capabilities, as it was shown for GPS/BDS [1,2,5,6,7], GPS/GLOANSS [3,8], and the four systems of GPS, Galileo, BDS and QZSS [9]. In this contribution, we will focus on the combination of three main satellite navigation systems: the U.S. Global Positioning System (GPS), the Chinese BeiDou navigation satellite system (BDS) and Russia’s GLObal NAvigation Satellite System (GLONASS), and investigate the AR and positioning performance by using only single-frequency observations from multi-GNSS.

The GPS and BDS use code division multiple access (CDMA) modulation, and signals from different satellites are transmitted on the same frequency. By contrast, GLONASS uses the frequency division multiple access (FDMA) modulation, which means signals from different satellite have different frequencies [8]. This FDMA modulation makes GLONASS ambiguity resolution rather difficult. One of the reasons is the inter-frequency bias (IFB) of phase observations caused by the analog hardware delay and the digital signal processing [10]. Different from GPS or BDS, IFB could not be eliminated in the double-differenced (DD) process, which prevents the GLONASS integer ambiguity resolution. Several approaches have been developed to estimate or calibrate the IFB [11,12,13]. In this research, we use the pre-calibrated IFB value for reliable ambiguity resolution. Another problem for GLONASS ambiguity resolution is that the wavelength of two inter-station single-differenced ambiguities is not equal, which means the GLONASS DD ambiguities cannot be formed like GPS. One method to deal with the different wavelengths of two single-differenced ambiguities in the DD observation equation is that phase observations are expressed in the unit of cycles instead of length. Although this strategy can recover the integer nature of the GLONASS DD ambiguities, receiver clock biases cannot be eliminated due to the different wavelengths [8,14]. Another method to tackle this problem is to estimate the single-differenced ambiguities and their covariance and then transform them to that of DD ambiguities for integer ambiguity resolution [15,16]. Comparative study has shown that these two methods have similar performance for GPS and GLONASS integration [17].

Although the multi-constellation concept brings benefits for GNSS positioning, the positioning performance of GNSS depends directly on the continuous tracking of the satellite signal [18,19]. Therefore, positioning methods based on GNSS observations alone are incapable of providing continuous navigation solution in GNSS-constrained environments. To overcome this issue, GNSS is often integrated with Inertial Navigation System (INS) [20,21,22,23,24,25], which can provide continuous position, velocity and attitude with high data rate when GNSS is not available. Besides, INS aiding plays an important role in ambiguity resolution [26,27,28] and cycle slip detection [29,30,31]. Meanwhile, the IMU sensor errors such as gyro drifts, accelerometer biases and scale factor errors can be estimated and compensated to further improve the navigation performance of INS [32,33,34,35]. Recently, several studies have focused on the integration of the combined GPS/BeiDou RTK and the INS using single- or dual-frequency data [36,37]. The results show that the probability of successful AR and positioning performance of the combined GPS/BDS/INS system can be improved significantly compared with the single GNSS system.

Compared with the high cost of dual-frequency RTK, it is more cost-effective to use the single-frequency receivers for positioning. Therefore, further efforts should be made to improve the positioning performance of single-frequency RTK. In this contribution, we tightly integrate GPS/BDS/GLONASS single-frequency RTK with MEMS-IMU to enhance the AR and positioning performance. The multi-GNSS single-frequency RTK model and tightly coupled strategy for multi-GNSS single-frequency RTK/INS integration is presented. A field vehicular test was conducted to assess the AR and positioning capabilities of the single-frequency GPS, GPS/BDS and GPS/BDS/GLONASS under different elevation cut-off angles varying from 15° to 40°.

This paper is organized as follows: Section 2 presents the mathematic model of multi-GNSS single-frequency RTK. Then, the integer ambiguity resolution method with INS aiding is briefly described in Section 3. Next, the implementation of the tightly-coupled integration of multi-GNSS RTK/INS is described in detail in Section 4. In Section 5, the collected data is processed and experimental results are compared and analyzed. Finally, some conclusions are given in Section 6.

## 2. Multi-GNSS SF-RTK Model

In general, the pseudoranges and carrier phase observation on $f_{1}$ frequency (herein GPS L1: 1575.42 MHz; BDS B1: 1562.098 MHz; GLONASS L1: 1602.006 +k× 0.5625 MHz, where k denotes the GLONASS satellite frequency number) are used together in the single-frequency RTK positioning. It is obvious that there is no frequency overlap among the three GNSS systems. It implies that system-specific reference satellite should be taken in the double-differencing (DD) formulation, i.e., one reference satellite per system.

The double-differenced GNSS pseudo-range and carrier phase for relative positioning can be expressed as follows in units of range, and we neglect time stamps for brevity:

(1) $$\nabla \Delta P=\nabla \Delta \rho +\nabla \Delta T+\nabla \Delta I+\nabla \Delta \varepsilon_{P}$$

(2) $$\lambda \nabla \Delta \varphi =\nabla \Delta \rho +\nabla \Delta T-\nabla \Delta I+\lambda \nabla \Delta N+\nabla \Delta \varepsilon_{\varphi }$$

where ∇Δ(⋅) denotes the double-differencing operator; P and φ are pseudo-range and carrier phase observations, respectively; ρ is the geometric distance in units of meters between the receiver and satellite; T and I are the tropospheric delay and ionospheric delay along the signal propagation path, respectively; λ and *N* represent carrier phase wavelength and integer ambiguity, respectively; $\varepsilon_{P}$ and $\varepsilon_{\varphi }$ are the measurement noise and unmodeled residual error (receiver noise, multipath, etc.) of pseudo-range and carrier phase, respectively. Specifically, for GLONASS FDMA signal structure, λ∇Δφ and λ∇ΔN could be expressed as follows:

(3) $$\lambda \nabla \Delta \varphi =\lambda^{k}\Delta \varphi^{k}-\lambda^{r}\Delta \varphi^{r}$$

(4) $$\lambda \nabla \Delta N=\lambda^{k}\Delta N^{k}-\lambda^{r}\Delta N^{r}$$

where the superscripts *k* and *r* denote the non-reference and reference satellite, respectively; Δ(⋅) is the inter-station single-differencing operator. In order to recover the integer nature of GLONASS DD ambiguities, we can rewrite Equation (4) as:

(5) $$\lambda \nabla \Delta N=\lambda^{k}\nabla \Delta N^{kr}+(\lambda^{k}-\lambda^{r})\Delta N^{r}$$

In the above equation, the single-differenced (SD) ambiguity of the reference satellite is present and it cannot be separated from DD ambiguities. One possible option to calculate the SD ambiguity is to use both SD pseudo-range and SD carrier phases [38]. In this research, we adopt the method proposed by Wang to search the optimal ambiguity in the DD observation equation [15].

It is well known that GNSS observables from low-elevation satellites suffer from larger atmospheric delay and multipath error compared with those high-elevation satellites [39]. The elevation-dependent weighting scheme [40] is adopted to determine the a priori variance of GNSS observations:

(6) $$\sigma^{2}=\{\begin{array}{l} \;\;\;\;\;\;\;\;\;\sigma_{0}^{2}\;,\;\;\;\;ele\geq \pi /6\\ {\left(\sigma_{0}/\sin(ele)\right)}^{2},\;\;else \end{array}$$

where $\sigma_{0}$ is the standard deviation (STD) at zenith, and ele is the elevation angle. A priori standard deviation for GPS, BDS and GLONASS carrier phase observation is set to 3 mm. The values for GPS, BeiDou IGSO/MEO, GLONASS and BeiDou GEO code observations are set to 0.35 m, 0.35 m, 0.5 m and 0.5 m, respectively.

For short baselines, the ionospheric and tropospheric delay in the DD observation Equations (1) and (2) are small enough, and can be neglected. Therefore, the unknown parameters in the DD observation equations are only the baseline increment vector and integer ambiguities. For multi-GNSS single-frequency RTK with GPS, BDS and GLONASS data, the equation to estimate the unknown parameters can then be written in matrix form as follows:

(7) $$\left[\begin{array}{c} \begin{array}{cccc} H^{G} & \Lambda^{G} & & \end{array}\\ \begin{array}{cccc} H^{C} & & \Lambda^{C} & \end{array}\\ \begin{array}{cccc} H^{R} & & & \Lambda^{R} \end{array} \end{array}\right]\left[\begin{array}{c} \delta p_{r}\\ \nabla \Delta N^{G}\\ \nabla \Delta N^{C}\\ \nabla \Delta N^{R} \end{array}\right]=\left[\begin{array}{c} \nabla \Delta L^{G}\\ \nabla \Delta L^{C}\\ \nabla \Delta L^{R} \end{array}\right]$$

where the superscripts ‘G’, ‘C’, ‘R’ represent GPS, BDS and GLONASS, respectively; ***H*** is the geometry matrix containing satellite-geometry information; $\delta p_{r}$ denotes the vector of baseline increment; Λ is the coefficient matrix of ambiguities; ∇ΔL is the DD Observed Minus Computed (OMC) vector.

In RTK positioning, the unknown coordinates of rover station are determined relative to a precisely known point using carrier phase observables. Correctly fixing the integer ambiguity parameter is a prerequisite to obtain centimeter-level positioning. GNSS ambiguity resolution generally involves three major steps. In the first step, the real-valued ambiguities and their corresponding variance-covariance (VC) matrix can be estimated using least-squares or a kalman filter. Then, the results from the first step are used to find the optimal integer solution, of which the least-squares ambiguity decorrelation adjustment (LAMBDA) method is widely used [41]. The final step is validation of the integer ambiguities, which is very crucial because incorrect AR will lead to unacceptable positioning error. The model-driven bootstrapped success rate and the data-driven ratio-test are usually used for ambiguity validation. The success rate indicates the underlying model strength and gives a quantitative assessment of the probability of correct integer estimation. The bootstrapped success rate is a sharp lower bound of the Integer Least-Squares (ILS) success rate with the following formula [42]:

(8) $$P_{s}=\prod_{i=1}^{n}\left(2\Phi \left(\frac{1}{2\sigma_{{\hat{N}}_{i|I}}}\right)-1\right)$$

where $\Phi (x)=\int_{-\infty }^{x}\frac{1}{\sqrt{2\pi }}\exp\left\{-\frac{1}{2}v^{2}\right\}dv$, and $\sigma_{{\hat{N}}_{i|I}}$ denotes the conditional standard deviations of the decorrelated ambiguities. However, the model-driven approach is not dependent on the real data, which means that the computed success rate can be very high even if the real measurements contain biases. Therefore, the data-driven index, ratio of the second minimum quadratic form of the integer ambiguity residuals to the minimum quadratic form of the residuals, is used together with the success rate in practice.

## 3. INS Aided Ambiguity Resolution Strategy

Carrier-phase based high-accuracy positioning based on carrier phase observation relies highly on the integer ambiguity resolution. However, it is very difficult or even impossible to resolve the integer ambiguities instantaneously for single-frequency GPS-only RTK, especially in dynamic environments. The situation becomes worse in urban environments where frequent signal blockages and multipath are present. A priori information from INS can be used to reduce the search space of integer ambiguities, and thus improving the efficiency and reliability of AR [43]. In this research, the INS-derived position vector will be used as a virtual measurement.

Assuming that the INS-derived position vector is $r_{INS}$, the linearized form of psedurange and carrier phase observation at this derived position can be expressed as:

(9) $$\left[\begin{array}{c} \varepsilon_{\rho }\\ \varepsilon_{\varphi } \end{array}\right]=\left[\begin{array}{cc} H & 0_{n\times n}\\ H & \lambda I_{n\times n} \end{array}\right]\left[\begin{array}{c} \delta p_{r}\\ \nabla \Delta N \end{array}\right]-\left[\begin{array}{c} \nabla \Delta P-\nabla \Delta r_{0}\\ \lambda \nabla \Delta \varphi -\nabla \Delta r_{0} \end{array}\right]$$

where n is the number of DD ambiguities and $\nabla \Delta r_{0}$ denotes the predicted DD pseudoranges computed with the INS-derived position. Then, the virtual observation from INS-derived position can be written as:

(10) $$\left[\varepsilon_{INS}\right]=\left[\begin{array}{cc} I_{3\times 3} & 0_{3\times n} \end{array}\right]\left[\begin{array}{c} \delta p_{r}\\ \nabla \Delta N \end{array}\right]-\left[0_{3\times 1}\right]$$

where $I_{3\times 3}$ is the identity matrix. If the weight matrices for the pseudorange, carrier phase and INS virtual measurement are $W_{\rho }$, $W_{\varphi }$ and $W_{INS}$, respectively, the coefficient matrix of the normal equation in the least-squares estimator can be obtained by combining Equations (9) and (10):

(11) $$\begin{array}{ll} N_{HH}=H_{BB}^{T}W_{\rho ,\varphi ,INS}H_{BB} & ={\left[\begin{array}{cc} H & 0_{n\times n}\\ H & \lambda I_{n\times n}\\ I_{3\times 3} & 0_{3\times n} \end{array}\right]}^{T}\;\left[\begin{array}{ccc} W_{\rho } & & \\ & W_{\varphi } & \\ & & W_{INS} \end{array}\right]\left[\begin{array}{cc} H & 0_{n\times n}\\ H & \lambda I_{n\times n}\\ I_{3\times 3} & 0_{3\times n} \end{array}\right]\\ & =\left[\begin{array}{cc} H^{T}W_{\rho }H+H^{T}W_{\varphi }H+W_{INS} & \lambda H^{T}W_{\varphi }\\ \lambda W_{\varphi }H & \lambda^{2}W_{\varphi } \end{array}\right] \end{array}$$

Then the least-squares solution of the unknown parameters with INS aiding can be written as

(12) $$X=N_{HH}^{-1}(H_{BB}W_{\rho ,\varphi ,INS}L)$$

where ***L*** is the Observed Minus Computed vector, which can be obtained from Equations (9) and (10). Compared with the coefficient matrix computed by using the pseduranges and carrier phases only, the term $W_{INS}$ in the top-left corner of Equation (11) can improve the strength of the normal equation, resulting in improved accuracy of the float ambiguities. The a priori constraint is crucial to single-epoch AR as the precision of pseudorange measurements is generally not enough for reliable AR, especially for single frequency data. Since INS can provide high-accuracy positioning information in short-term period, this strong constraint will definitely improve the probability of correctly fixing the ambiguities. Once the float ambiguities and their variance-covariance are obtained, the fixed ambiguities can be resolved and validated using the method described in the previous section.

## 4. Implementation of Tightly-Coupled Multi-GNSS SF-RTK/INS Integration

In this contribution, the tightly-coupled integration of GPS/BDS/GLONASS single-frequency RTK/MEMES-IMU is implemented using an extended kalman filter (EKF). The EKF fuses measurements from the GNSS and IMU to obtain an optimal estimate of the system states. The system models and measurement models of the EKF are given below. The system models include the INS dynamic model and the IMU sensor uncertainty model [33].

### 4.1. INS Danymical Model

In this research, the attitude errors are expressed in terms of ψ-angle, which is called the ψ-angle error model [44]. In the ψ-angle error model, the INS error analysis is done with respect to the c-frame (locally levelled at the computed position):

(13) $$\begin{array}{c} \delta {\dot{r}}^{c}=-\omega_{ec}^{c}\times \delta r^{c}+\delta v^{c}\\ \delta {\dot{v}}^{c}=f^{c}\times \psi -(2\omega_{ie}^{c}+\omega_{ec}^{c})\times \delta v^{c}+\delta g^{c}+C_{b}^{p}\delta f^{b}\\ \dot{\psi }=-(\omega_{ie}^{c}+\omega_{ec}^{c})\times \psi -C_{b}^{p}\delta \omega_{ib}^{b} \end{array}$$

where $\delta \dot{r}$, $\delta \dot{v}$, $\dot{\psi }$ are the derivative of position, velocity and attitude error vectors, respectively; $\omega_{ie}^{c}$ is the angular rate of e-frame (i.e., Earth-centered earth-fixed, ECEF) with respect to the i-frame, projected to the c-frame; $\omega_{ec}^{c}$ is the angular rate of c-frame with respect to e-frame, projected to c-frame; $\delta g^{c}$ is the gravity error vector projected in the c-frame; $\delta f^{b}$, $\delta \omega_{ib}^{b}$ are the inertial sensor errors; $C_{b}^{p}$ is the rotation matrix from the b-frame (i.e., Forward-Right-Down, FRD) to the platform frame.

### 4.2. IMU Sensor Uncertainty Model

Accurate modeling of the MEMS IMU errors is of great importance to improve the navigation performance. Therefore, the IMU errors including both gyroscope and accelerometer errors are augmented into the extended kalman filter states and estimated on-line in this paper. The bias error and scale factor error of the IMU are generally modeled as first-order Gauss-Markov (GM) processes in most kalman filter implementation for GPS/INS integration algorithms [45], which can be expressed as follows:

(14) $$\left(\begin{array}{c} \delta \dot{b}\\ \delta \dot{s} \end{array}\right)=\left(\begin{array}{c} -\frac{1}{\tau_{b}}\delta b\\ -\frac{1}{\tau_{s}}\delta s \end{array}\right)+w$$

where δb, δs denote the bias errors and scale factor errors, respectively, both including gyroscope error and accelerometer error; $\tau_{b}$, $\tau_{s}$ are the corresponding correlation time of the random process; w is the driving white noise. It should be noted that the correlation time related to the bias and scale factor errors of the gyroscope and accelerometers may be different and these parameters can be obtained using the method of Allan variance.

Therefore, the whole system model for continuous EKF by combining the ψ-angle error model and the IMU sensor error model can be described as:

(15) $${\dot{X}}_{INS}=FX_{INS}+Gw$$

where ***F*** is the dynamic matrix and its expression can be found in [46]; G is the noise distribution matrix. The state vector ***X*** can be written as:

(16) $$X={\left[\begin{array}{cccc} \begin{array}{cccc} {\left(\delta r^{c}\right)}^{T} & {\left(\delta v^{c}\right)}^{T} & \psi^{T} & b_{g}^{T} \end{array} & b_{a}^{T} & s_{g}^{T} & s_{a}^{T} \end{array}\right]}^{T}$$

where $\delta r^{c}$ and $\delta v^{c}$ can be written as follows:

(17) $$\delta r^{c}={\left[\begin{array}{ccc} \delta r_{N} & \delta r_{E} & \delta r_{D} \end{array}\right]}^{T}$$

(18) $$\delta v^{c}={\left[\begin{array}{ccc} \delta v_{N}^{c} & \delta v_{E}^{c} & \delta v_{D}^{c} \end{array}\right]}^{T}$$

ψ denotes the attitude errors; $b_{g}$ is the gyro bias error; $b_{a}$ is the accelerometer bias error; $s_{g}$ and $s_{a}$ represent the scale factor errors of gyro and accelerometer, respectively. 

### 4.3. Multi-GNSS SF-RTK/INS Measurement Model

When both the GNSS observables and IMU data arrive at the same epoch, the tightly-coupled RTK/INS measurement model will be used to update the EKF. The measurement model for EKF in the discrete-time domain is expressed as:

(19) $$Z_{k}=H_{k}X_{k}+\eta_{k}$$

where $H_{k}$ is the design matrix, and $Z_{k}$ is the measurement vector whose value are the differences between INS-predicted measurement and GNSS raw observables:

(20) $$Z_{k}=\left[\begin{array}{c} \nabla \Delta {\hat{\rho }}_{INS}-\nabla \Delta P_{GNSS}\\ \nabla \Delta {\hat{\rho }}_{INS}-\lambda \nabla \Delta \varphi_{GNSS} \end{array}\right]$$

where the subscripts “INS” and “GNSS” represent the predicted GNSS measurements using INS-derived information and observables from the GNSS receivers, respectively. The INS-predicted ranges are calculated using the INS-updated solution and the position of the satellites. The linearized form of INS-predicted DD observation can be written as:

(21) $$\nabla \Delta \hat{\rho }=\nabla \Delta \rho +({\vec{e}}_{k}-{\vec{e}}_{j})\cdot \delta r^{e}$$

where $\vec{e_{k}}$ and $\vec{e_{j}}$ are the unit line-of-sight (LOS) vectors from the IMU center to the *k*th and *j*th satellite, respectively; $\delta r^{e}$ is the position error vector expressed in the e-frame.

Since the IMU and the GNSS antenna are not at the same place in the vehicle, the position of IMU center is different from that of the GNSS antenna phase center, which is called the lever-arm effect. The lever-arm correction in the n-frame (i.e., North-East-Down, NED) can be described as:

(22) $$r_{GNSS}^{n}=r_{IMU}^{n}+D_{R}^{-1}C_{b}^{n}\ell_{GNSS}^{b}$$

(23) $$D_{R}^{-1}=\left[\begin{array}{ccc} \frac{1}{R_{M}+h} & 0 & 0\\ 0 & \frac{1}{(R_{N}+h)\cos\varphi } & 0\\ 0 & 0 & -1 \end{array}\right]$$

where $r_{GNSS}^{n}$ and $r_{IMU}^{n}$ are geodetic coordinates for the GNSS receiver and IMU center, respectively; $C_{b}^{n}$ denotes the rotation matrix form the b-frame to the n-frame; $\ell_{GNSS}^{b}$ is the lever-arm vector in the b-frame. In this research, the lever arm between the IMU and the GNSS antenna was accurately measured to the millimeter level.

The computed position of the GNSS antenna phase center based on the true model in (22) can be written as follows:

(24) $$\begin{array}{ll} {\hat{r}}_{GNSS}^{n} & ={\hat{r}}_{IMU}^{n}+D_{R}^{-1}{\hat{C}}_{b}^{n}\ell_{GNSS}^{b}\\ & =r_{IMU}^{n}+D_{R}^{-1}\delta r_{IMU}^{n}+D_{R}^{-1}[I-(\phi \times )]C_{b}^{n}\ell_{GNSS}^{b}\\ & =r_{GNSS}^{n}+D_{R}^{-1}\delta r_{IMU}^{n}+D_{R}^{-1}[(C_{b}^{n}\ell_{GNSS}^{b})\times ]\phi \end{array}$$

The position uncertainty term of GNSS and IMU can be further expressed as:

(25) $$\begin{array}{ll} \delta r_{GNSS}^{n} & =\delta r_{IMU}^{n}+[(C_{b}^{n}\ell_{GNSS}^{b})\times ]\phi \\ & =\delta r_{IMU}^{n}+[(C_{b}^{n}\ell_{GNSS}^{b})\times ](\psi +\delta \theta )\\ & \approx \delta r_{IMU}^{n}+[(C_{b}^{n}\ell_{GNSS}^{b})\times ]\psi \end{array}$$

where the term $[(C_{b}^{n}\ell_{GNSS}^{b})\times ]\delta \theta$ is very small and can be neglected.

Then the design matrix $H_{k}$ in Equation (19) can be derived from (1), (2), (20), (21), and (25), which can be written as:

(26) $$H_{k}=\left[\begin{array}{c} \begin{array}{cccc} H_{\rho }^{*}\cdot C_{n}^{e} & 0_{n\times 3} & H_{\rho }^{*}\cdot C_{n}^{e}\cdot [(C_{b}^{n}\ell_{GNSS}^{b})\times ] & \begin{array}{c} 0_{n\times 12} \end{array} \end{array}\\ \begin{array}{cccc} H_{\varphi }^{*}\cdot C_{n}^{e} & 0_{n\times 3} & H_{\varphi }^{*}\cdot C_{n}^{e}\cdot [(C_{b}^{n}\ell_{GNSS}^{b})\times ] & \begin{array}{c} 0_{n\times 12} \end{array} \end{array} \end{array}\right]$$

where “*” represents “G” for GPS, “C” for BDS, and “R” for GLONASS; $C_{n}^{e}$ denotes the rotation matrix from the n-frame to the e-frame; n is the whole number of DD observations.

**Figure 1:**
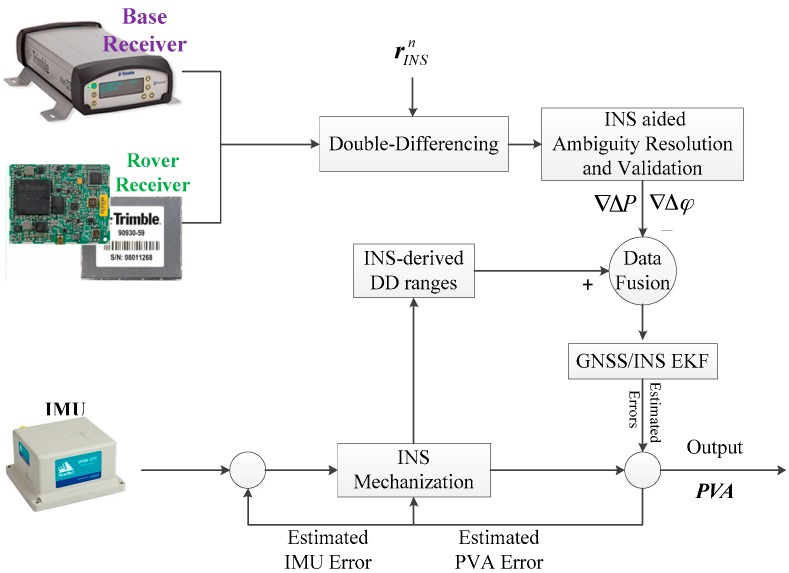
Architecture of the tightly-coupled integration of multi-GNSS SF-RTK/INS (Note: PVA represents the position, velocity and attitude information).

An overview of the tightly-coupled integration of GPS/BDS/GLONASS single-frequency RTK/MEMS-IMU algorithm is shown in Figure 1. After initialization of the tightly-coupled algorithm, the compensated raw IMU data is used to provide the continuous navigation result (position, velocity, and attitude) through INS mechanization. When the GNSS data are available, the pseudorange and carrier phase DD measurements are formed from base and rover receivers. The INS-aided AR and validation module use the proposed strategy in Section 3 to fix the float ambiguities. Then, the EKF of the tightly-coupled integration fuses DD pseudorange and carrier phase observations with the INS-predicted DD ranges to optimally estimate the error states and covariance. Finally, the estimated IMU sensor errors (i.e., the bias and scale factor of gyroscope and accelerometer) are fed back to correct the raw IMU output. Meanwhile, the navigation solution of INS mechanization is corrected with the estimated position, velocity and attitude errors. In the tightly-coupled integration, the INS-only mode and the Kalman filter time prediction mode are more frequent than the Kalman filter measurement update mode due to the higher sampling rate of IMU. If no GNSS data is available, the IMU data will be processed epoch-by-epoch. Otherwise, the tightly-coupled measurement model will be applied to update the state vector and the corresponding covariance matrix of the Kalman filter.

## 5. Experimental Validation and Discussion

**Figure 2:**
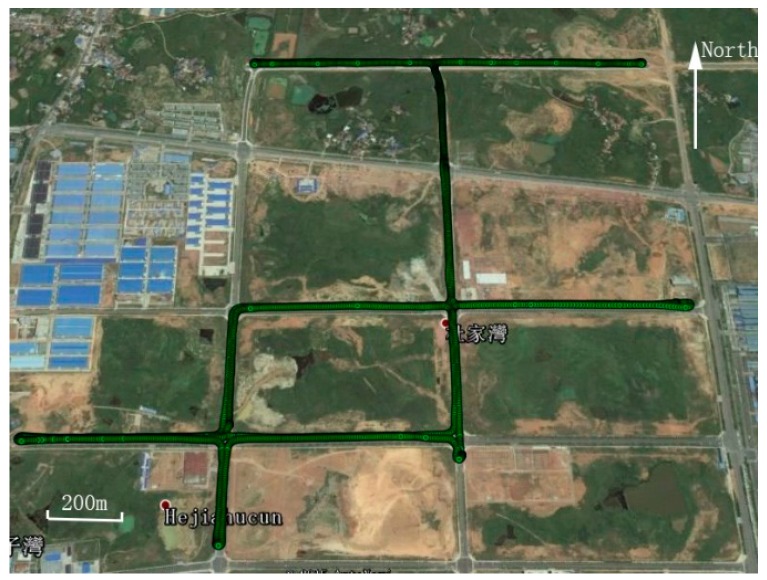
Vehicular trajectory of the field test.

**Figure 3:**
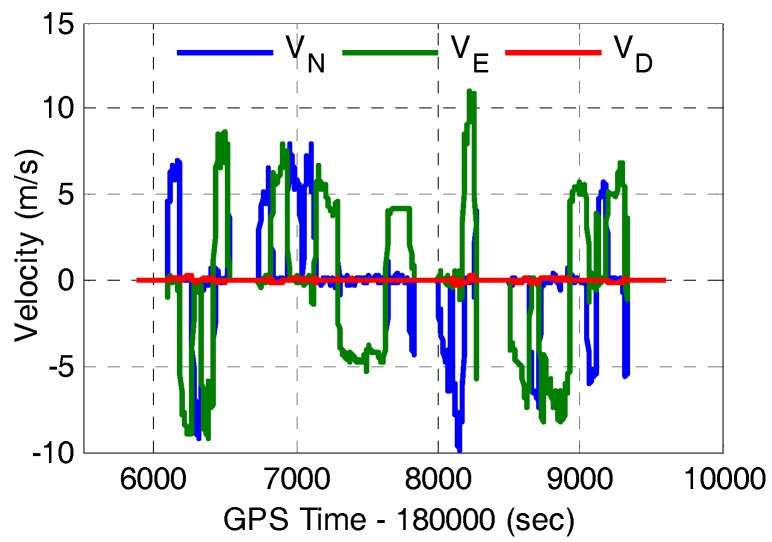
Velocity of the vehicle in the field test.

In order to evaluate the positioning performance of the proposed tightly-coupled integration of multi-GNSS single-frequency RTK and INS, a field vehicular test around Wuhan City in China was conducted. Several integrated navigation systems were mounted on the test vehicle, among which the data from SPAN-CPT was processed and analyzed in this paper. The SPAN-CPT is provided by NovAtel Inc. (Calgary, AB, Canada), and it consists of a MEMS-based IMU and a NovAtel OEM4 receiver. The performance specifications of the IMU sensor is shown in Table 1. The receiver of base station is a Trimble NetR9 multi-GNSS receiver (Sunnyvale, CA, USA). The rover station consists of a Trimble BD930 OEM receiver. The baseline separation is less than 2km, and thus the ionospheric and tropospheric delay can be neglected in the data processing. In the field test, the sampling rate of the raw IMU data and the raw GNSS data from base and rover GNSS receivers are 100 Hz and 1 Hz, respectively. The time span is about 1 h and the corresponding trajectory is shown in Figure 2. Figure 3 shows the velocity of the vehicle in the field test. It can be seen that there are frequent tuning and acceleration in the horizontal direction.

### 5.1. Satellite Availability and Position Dilution of Precision

**Figure 4:**
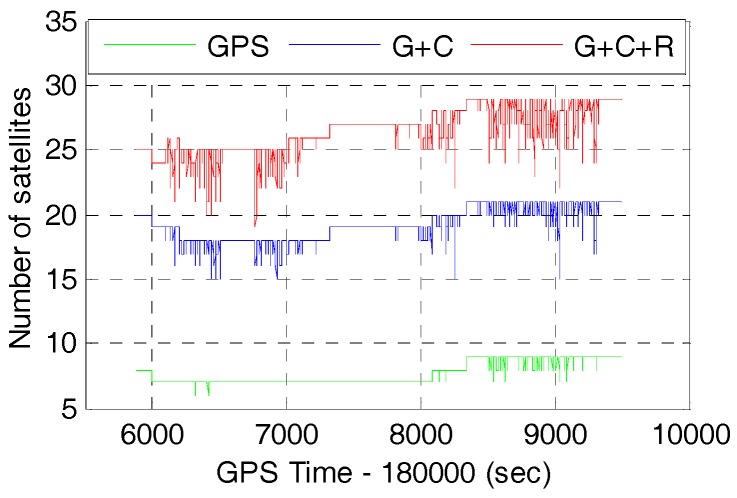
Number of satellites of GPS (G), GPS/BDS (G+C), GPS/BDS/GLONASS (G+C+R) with 15° cut-off elevation angle.

**Figure 5:**
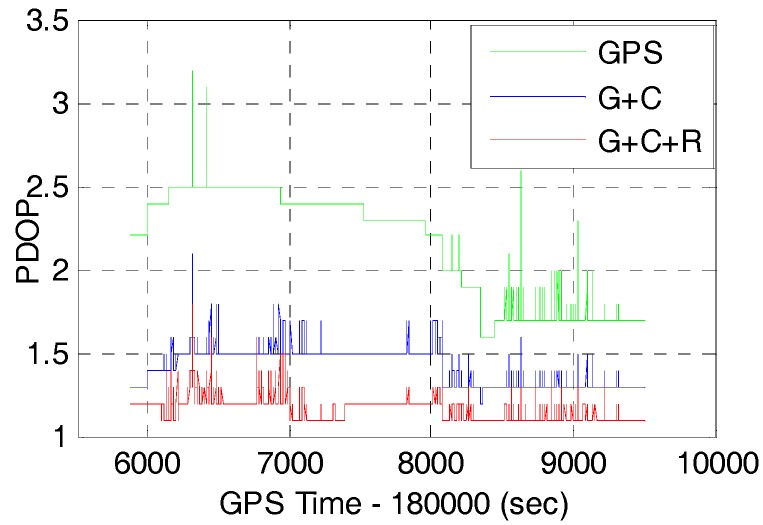
PDOP of GPS (G), GPS/BDS (G+C), GPS/BDS/GLONASS (G+C+R) with 15° cut-off elevation angle.

The available GNSS satellites and PDOP will be investigated firstly as they are crucial factors for GNSS positioning accuracy. The number of available satellites and the corresponding Position Dilution of Precision (PDOP) of GPS, GPS/BDS (G+C), and GPS/BDS/GLONASS (G+C+R) with 15° cut-off elevation angle are shown in Figure 4 and Figure 5, respectively. 

It can be seen that the number of satellites are increased significantly by adding the BDS and GLONASS to the GPS. Statistics from Figure 4 and Figure 5 show that the average number of satellites of GPS, GPS/BDS, and GPS/BDS/ GLONASS are 7.7, 19.2 and 26.3, and the corresponding PDOP values are 2.14, 1.42 and 1.17, respectively. In comparison with the GPS, the PDOP improvements of GPS/BDS and GPS/BDS/GLONASS are about 33.6% and 45.3%, respectively. It is expected that better AR performance and positioning performance could be achieved for single-frequency users with multi-GNSS observations.

**Figure 6:**
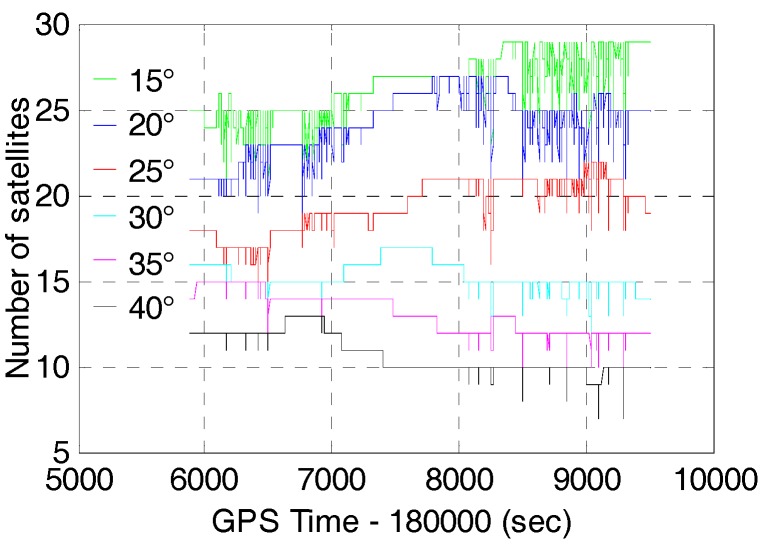
Number of Satellites of GPS/BDS/GLONASS (G+C+R) with cut-off elevation angle ranging between 15° and 40°.

**Figure 7:**
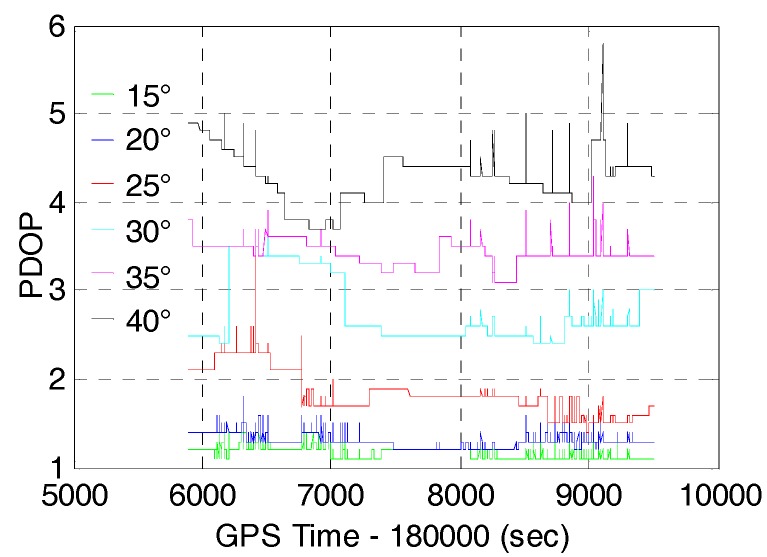
PDOP of GPS/BDS/GLONASS (G+C+R) with cut-off elevation angle ranging between 15° and 40°.

Since multi-GNSS can provide more available satellites for positioning, the GNSS applicability is expected to be improved correspondingly in constrained environment such as urban canyons. In order to evaluate the AR and positioning performance of multi-GNSS in constrained environment, we processed the data for six different cut-off elevation angles ranging between 15° and 40° with an interval of 5°.The number of satellites and PDOP of GPS/BDS/GLONASS with cut-off elevation angles ranging between 15° and 40° are shown in Figure 6 and Figure 7, respectively. The number of satellites decrease from 26.3 to 10.8 and the corresponding PDOP becomes larger (from 1.17 to 4.28) with the cut-off elevation angles ranging from 15° to 40°. Figure 4 and Figure 6 show that the number of satellites of GPS/BDS/GLONASS at 40° cut-off elevation angle is larger than that of the GPS at 15° cut-off elevation angle for most of the time. However, the corresponding PDOP of the GPS/BDS/GLONASS is about two times larger than that of the GPS. It is due to the large VDOP (Vertical Dilution of Precision) caused by the distribution of satellites with high elevation. Table 2 shows the mean DOPs of GPS/BDS/GLONASS for 15° to 40° cut-off elevation angles. 

Obviously, the mean Horizontal Dilution of Precision (HDOP) of the GPS/BDS/GLONASS at 40° cut-off elevation angle is only 1.1521, but the VDOP is nearly four times larger. Therefore, the positioning performance of multi-GNSS may be degraded to some extent in vertical direction with higher cut-off elevation angles.

### 5.2. Single-Epoch AR Performance for Different System Configurations

Correct integer ambiguity resolution is a prerequisite for differential carrier-phase based centimeter-level positioning. Here, we focus on the single-epoch ambiguity resolution as this is the most challenging case. In order to evaluate the single-frequency AR performance, six different system configurations, including the RTK of GPS, GPS/BDS, GPS/BDS/GLONASS and the corresponding tightly-coupled RTK/INS integration, were used to process the experimental data. The AR performance in terms of ratio values from the AR process and the empirical success rate would be compared and analyzed. According to the results presented in [2], the AR of the combined GNSS system is more reliable due to the better satellite geometry, and thus the threshold value for the combined GNSS system can be set to a slightly smaller value than the single GNSS system. In our data analysis, the predefined success rate is set to 0.99, and the critical ratio value is set to 3.0 for the GPS and 2.0 for the combined GPS/BDS or GPS/BDS/GLONASS system.

**Figure 8:**
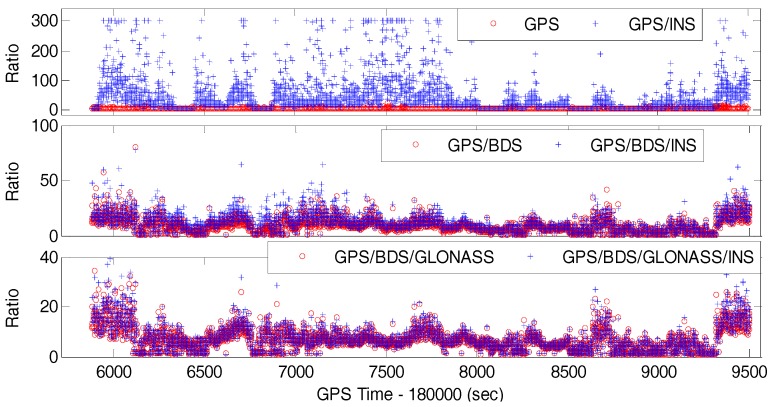
Ratio values of single-epoch AR for different system configurations (15° cut-off elevation angle).

The ratio-test value can be used to indicate the probability of correctly fixing the integer ambiguities [47]. Figure 8 shows the time series of ratio values for six different system configurations for an elevation cut-off angle of 15°. In the case of GPS only, the ratio values are mostly less than the critical value of three, which means that most of the ambiguities cannot pass the validation test and they have a low probability to be correctly resolved. By comparison, the ratio values obtained from either GPS/BDS or GPS/BDS/GLONASS are large enough to pass the validation test in most of the time. This is one of the benefits from multi-GNSS due to the greatly increased satellites and geometric strength. Besides, the ratio values increase significantly after the inclusion of IMU data, especially for the case of GPS/INS integration. Hence, a priori position constraint from INS indeed enhances the probability of correctly fixing the integer ambiguities.

We also investigate the empirical success rate of single-epoch AR for different system configurations with elevation cut-off angles of 15, 20, 25, 30, 35 and 40°. The resolved ambiguities in single-epoch mode will be compared with the reference ambiguities to ensure the correctness of the fixed ambiguities. Only if all the DD ambiguities from one observation epoch are correctly fixed, this epoch will be considered as a fixed epoch. The reference ambiguities were estimated by using dual-frequency GPS/BDS/GLONASS data and assuming the time-constant property of the ambiguities. The empirical success rate can be defined as:

(27) $$P_{SE}=\frac{\mathrm{number}\;\mathrm{of}\;\mathrm{correctly}\;\mathrm{fixed}\;\mathrm{epochs}}{\mathrm{total}\;\mathrm{number}\;\mathrm{of}\;\mathrm{epochs}}$$

Therefore, it directly reflects the availability of ambiguity-resolved positioning. Table 3 shows the empirical single-epoch success rate for different system configurations with 10°–40° cut-off elevation angles, and the RMSs of the corresponding number of satellites are shown within parentheses. 

Obviously, the greatly increased satellites from multi-GNSS bring significant advantates to AR. As one would expect, the GPS-only system has a very low success rate only at 8.4% with 15° elevation cut-off angle and the success rate get much smaller as cut-off elevation gets larger, which indicates that single-frequency GPS-only AR is not possible instantaneously. By comparison, the success rates of GPS/BDS and GPS/BDS/GLONASS RTK are 86.4 and 84.3% at 15° elevation cut-off angle, respectively. The success rate of the combined GPS/BDS/GLONASS is slightly worse than that of the GPS/BDS, which may be due to the inter-channel code bias of GLONASS satellites and multipath effects. As the cut-off elevation gets larger, the success rate of GPS/BDS reaches the top level (92.7%) at 25° elevation cut-off angle, and the figure for GPS/BDS/GLOLNSS is 95.9% at 35° elevation cut-off angle. Moreover, the success rate of GPS/BDS/GLONASS at 40° elevation cut-off angle is 50.3%, and it is almost double that of the GPS/BDS. Apparently, the multi-GNSS shows significant advantages, especially in the case of higher cut-off elevation angles.

The results from the table also show that the single-epoch single-frequency AR performance can be further improved with inertial aiding. The empirical success rate of GPS/INS has increased by about eight times, arriving at 77.7% at 15° cut-off elevation angle. However, the figure for the tightly-coupled integration of GPS/BDS/INS and GPS/BDS/GLONASS/INS shows only a little more improvement than that of the corresponding RTK solutions when the cut-off elevation angle is below 30°. The main reason for this is that the available satellites and geometric strength are enough for AR in this case, and some observations with low-elevation multipath make the AR difficult. As the cut-off elevation angle gets larger to 30° or 40°, the success rates of both GPS/BDS/INS and GPS/BDS/GLONASS/INS are over 99%, showing significant improvement in comparison with the corresponding GNSS RTK solution. It seems that inertial aiding can make up the drawback of the GNSS-only system especially when the GNSS operates in challenged situations like the multi-GNSS RTK with higher elevation cut-off angle. It is of great importance because the GNSS applicability can be improved significantly in constrained environment, such as urban canyons.

### 5.3. Positioning Performance of RTK and the Tightly-Coupled RTK/INS Integration

In order to evaluate the positioning performance of RTK and the tightly-coupled RTK/INS integration, the field data was processed in GPS, GPS/BDS, and GPS/BDS/GLONASS RTK mode and the corresponding tightly-coupled integration mode. In the data processing, we used the “fix and hold” mode proposed by Takasu and Yasuda [16] to fix the ambiguities such that almost all the epochs can be correctly fixed. This mode will impose a tight constraint on the fixed ambiguities from the previous epoch, and this constraint is treated as a “pseudo”-measurement in the estimation of real-valued ambiguity. In this way, the impacts of multi-GNSS on the position accuracy can be evaluated. The post-processed solution of the dual-frequency tightly-coupled GPS/BDS/GLONASS/ INS with fixed ambiguities was used as the “reference trajectory” to assess the positioning performance.

**Figure 9:**
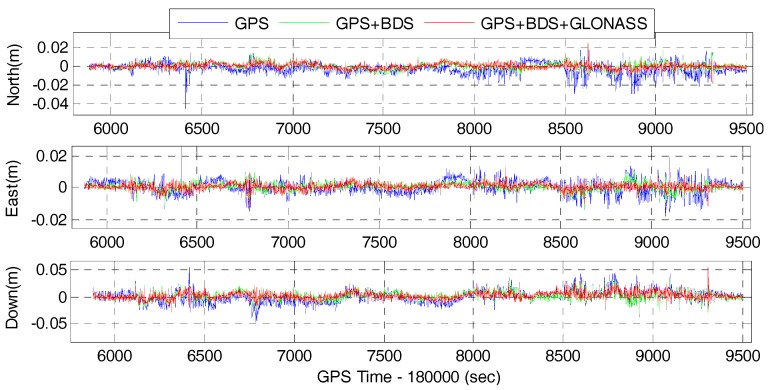
Position difference of RTK for GPS, GPS/BDS, GPS/BDS/GLONASS at 15° cut-off elevation angle.

**Figure 10:**
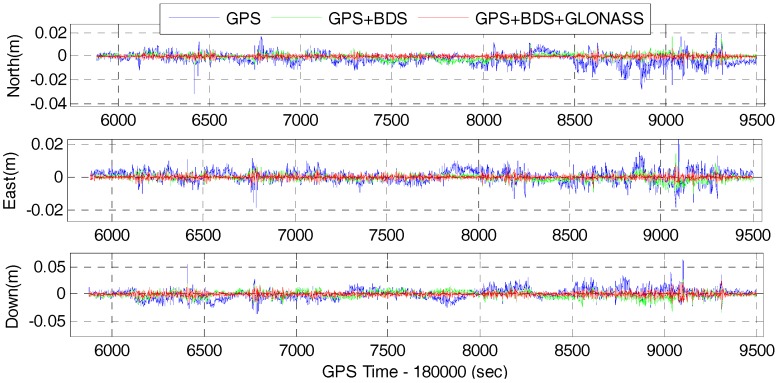
Position difference of RTK/INS for GPS, GPS/BDS, GPS/BDS/GLONASS at 15° cut-off elevation angle.

The time series of position difference of the GPS, GPS/BDS, GPS/BDS/GLONASS RTK and the corresponding tightly-coupled RTK/INS integration with 15° cut-off elevation angle are depicted in Figure 9 and Figure 10, respectively. It can be seen that the precision of the fixed solutions are comparable to each other for the GPS, GPS/BDS, and GPS/BDS/GLONASS. However, the positioning difference for GPS/BDS/GLONASS are much more stable than that of the GPS system, and slightly better than the combined GPS/BDS system in north, east and vertical directions. This visible improvements are mainly due to the greatly increased satellites and better geometric structure of multi-GNSS constellations.

Table 4 shows the RMS of the position difference of RTK and RTK/INS for GPS, GPS/BDS, and GPS/BDS/GLONASS at 15° cut-off elevation angle with respect to the reference solution. The statistics indicate that the RMS values of GPS RTK are significantly improved from 5.8, 4.0, and 9.9 mm to 2.8, 2.2, 6.8 mm of GPS/BDS/GLONASS RTK with improvements of 51.7, 45.0 and 31.3% in the north, east and vertical directions, and the improvements for the GPS/BDS RTK are 43.1, 40.0 and 26.3%. The results also indicate that the tightly-coupled RTK/INS integration can further improve the positioning performance compared with the GNSS RTK solutions. The RMS values of the tightly-coupled GPS RTK/INS are 5.0, 3.4 and 8.9 mm with 16.0, 15.0 and 10.1% improvements in north, east and vertical directions, and the improvements for GPS/BDS/INS are 33.3, 29.2 and 26.0%. Among which, the tightly-coupled GPS/BDS/GLONASS RTK/INS integration can achieve the best performance with the RMS of 1.2, 1.2 and 3.6 mm in the north-east-down directions. Similar to the GNSS RTK solutions, the results also show that there are significant improvements in terms of position by using the tightly-coupled multi-GNSS RTK/INS integration in comparison with the GPS RTK/INS integration. The improvements are 56.0, 50.0 and 39.3% for GPS/BDS RTK/INS integration in north, east and vertical components, and the figure for GPS/BDS/GLONASS RTK/INS integration are 76.0, 64.7 and 59.6%. Therefore, the single-frequency positioning performance can be enhanced indeed by combining the multi-GNSS and IMU data.

**Figure 11:**
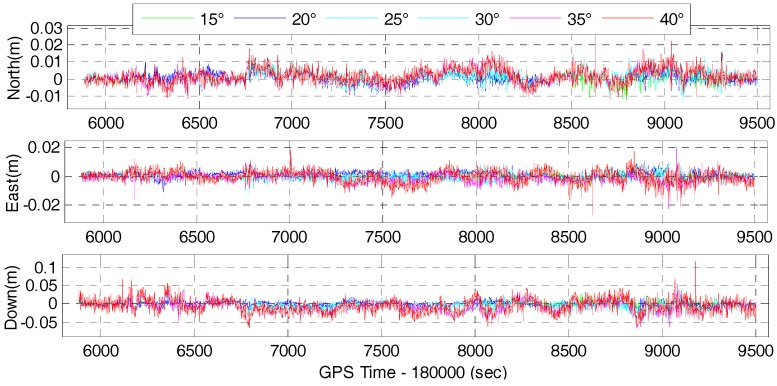
Position difference of GPS/BDS RTK for elevation cut-off angles of 15, 20, 25, 30, 35 and 40°.

**Figure 12:**
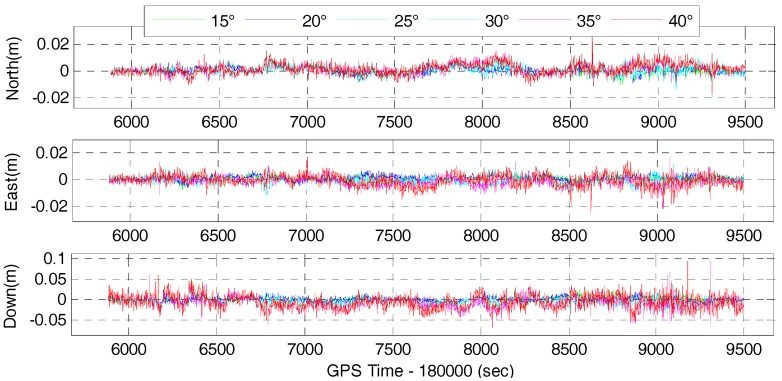
Position difference of GPS/BDS/GLONASS RTK for elevation cut-off angles of 15, 20, 25, 30, 35 and 40°.

We also investigate the single-frequency multi-GNSS positioning performance for different elevation cut-off angles to simulate situations in GNSS constrained-environments or when low-elevation multipath is to be avoided. Therefore, the field data was also processed in GPS/BDS and GPS/BDS/GLONASS RTK mode and the corresponding tightly-coupled RTK/INS integration mode with elevation cut-off angles of 15, 20, 25, 30, 35 and 40°. The time series of positioning difference of GPS/BDS and GPS/BDS/GLONASS RTK with respect to the reference solution are depicted in Figure 11 and Figure 12, respectively. It is clear that the position difference is within 2 cm in north and east directions for both the GPS/BDS and GPS/BDS/GLONASS RTK, and the vertical component is within 5 cm except for a few epochs. It indicates that the comparable accuracy can be achieved under 40° elevation cut-off angle for multi-GNSS positioning if the ambiguities are resolved to their true value. Obviously, the time series of positioning difference with 40° elevation cut-off angle are not as stable as those with lower elevation cut-off angle, especially in the vertical directions.

**Figure 13:**
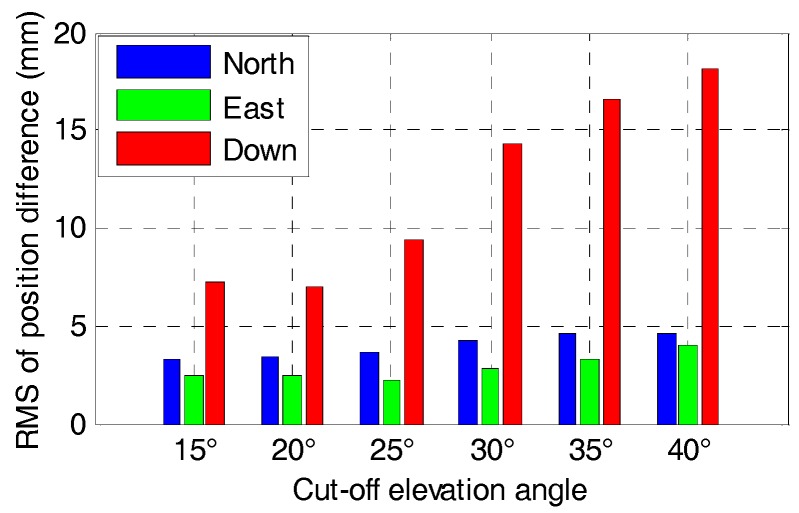
RMS of positioning difference of RTK for GPS/BDS with elevation cut-off angles of 15, 20, 25, 30, 35 and 40°.

**Figure 14:**
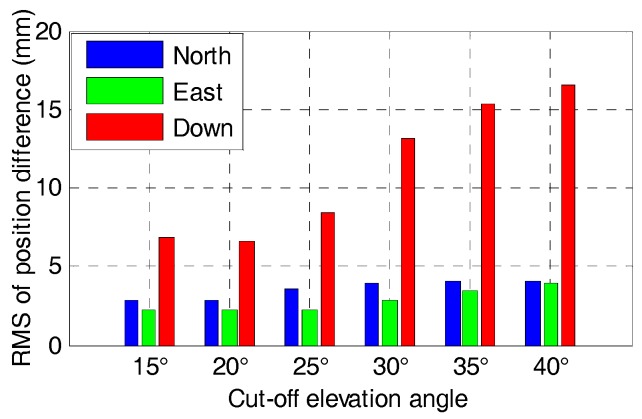
RMS of positioning difference of RTK for GPS/BDS/GLONASS with elevation cut-off angles of 15, 20, 25, 30, 35 and 40°.

The statistics in terms of RMS of the positioning difference for GPS/BDS and GPS/BDS/GLONASS RTK with elevation cut-off angles of 15, 20, 25, 30, 35 and 40° are shown in Figure 13 and Figure 14, respectively. The RMS of the position difference for GPS/BDS RTK drops from 3.3, 2.4 and 7.3 mm to 4.6, 4.4 and 18.1 mm in north-east-down directions when the elevation cut-off angle increases from 15 to 40°. The figures for GPS/BDS/GLONASS RTK are 2.8, 2.2 and 6.8 mm with increments of 15.15, 8.33 and 6.85% to 4.1, 4.0 and 16.6 mm with increments of 10.87, 9.09 and 8.29%. Clearly, all the RMS values of the position difference are under 5 mm in the north and east directions for elevation cut-off angles ranging from 15 to 40°, and the RMS value of the vertical component begins to increase gradually when the elevation cut-off angle is above 25°. From the DOPs listed in Table 2, we know that the HDOP changes very slowly and still has a small value even with 40° elevation cut-off angle for GPS/BDS/GLONASS. Hence, the positioning results are consistent with the DOP values.

**Figure 15:**
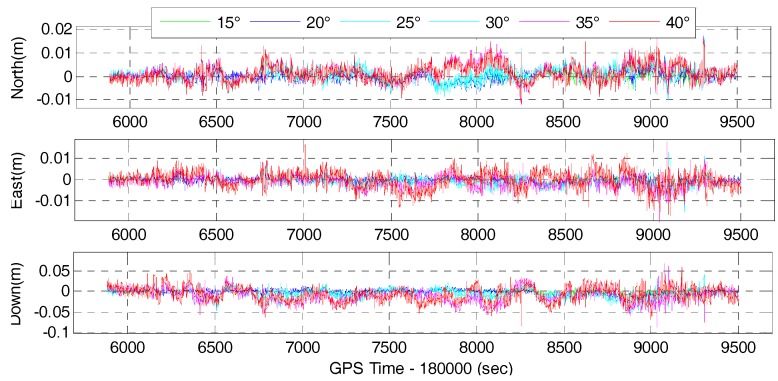
Position difference of GPS/BDS/INS with elevation cut-off angles of 15, 20, 25, 30, 35 and 40°.

**Figure 16:**
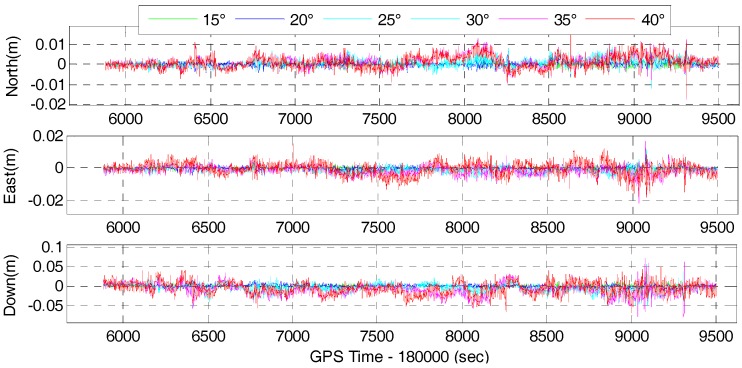
Position difference of GPS/BDS/GLONASS/INS with elevation cut-off angles of 15, 20, 25, 30, 35 and 40°.

The time series of the position difference for the corresponding tightly-coupled GPS/BDS/INS and GPS/BDS/GLONASS/INS integration with elevation cut-off angles of 15, 20, 25, 30, 35 and 40° are shown in Figure 15 and Figure 16, respectively. 

**Figure 17:**
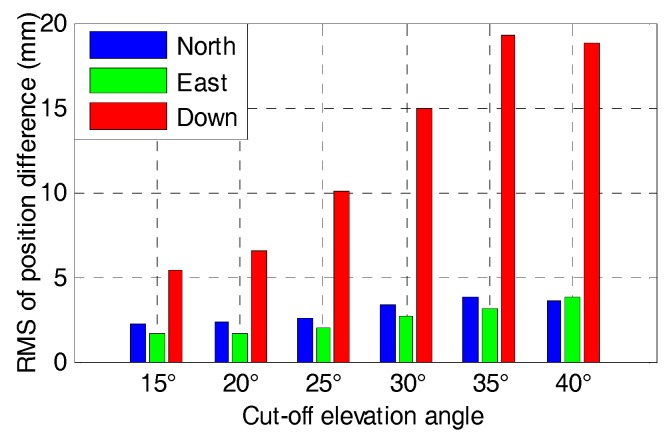
RMS of positioning difference of GPS/BDS/INS with elevation cut-off angles of 15, 20, 25, 30, 35 and 40°.

**Figure 18:**
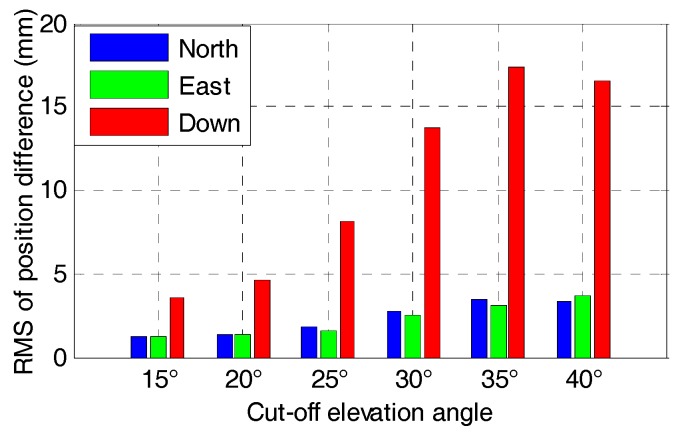
RMS of positioning difference of GPS/BDS/GLONASS/INS with elevation cut-off angles of 15, 20, 25, 30, 35 and 40°.

It can be found that the time series of the tightly-coupled integration is very similar to the corresponding GNSS RTK solution. Apparently, the time series of the tightly-coupled GPS/BDS/GLONASS RTK/INS integration is more stable than that of the GPS/BDS/INS integration, especially for higher elevation cut-off angles. Figure 17 and Figure 18 show the RMS of the position difference for GPS/BDS/INS and GPS/BDS/GLONASS/INS integration with different elevation cut-off angles, respectively. 

The RMS of the tightly-couple GPS/BDS/INS integration drops from 2.2, 1.7, and 5.4 mm to 3.6, 3.6 and 18.8 mm in north-east-down components, and the figures for GPS/BDS/GLONASS/INS integration are 1.2, 1.2 and 3.6 mm to 3.3, 3.7 and 16.6 mm. We also notice that the RMS of the tightly-coupled GPS/BDS/GLONASS/INS integration at 25° elevation cut-off angle is still better than that of the GPS-only solution (Table 4) in the north, east and vertical directions. Even if the elevation cut-off angle is set to 40°, the tightly-coupled GPS/BDS/GLONASS/INS integration still has very good positioning performance with only a little accuracy loss in the vertical direction.

### 5.4. INS Bridging Capabilities During GNSS Outages

As shown in the previous section, single frequency multi-GNSS RTK users can obtain high-precision positioning performance under open-sky conditions with good satellite availability. However, big positioning errors or even no position output will occur frequently in GNSS-challenged environments due to the multipath effect and signal blockages. However, the capability to maintain the continuous positioning performance is of great importance for some kinematic positioning applications. Besides, the rapid recovery of AR after GNSS outages is critical for the recovery of precise positioning, which also improves the system availability. It is expected that rapid recovery of AR can be achieved with the help of INS.Therefore, we also analyze the position drift errors with short-term GNSS signal outages and the recovery performance of AR after the end of GNSS outages.

**Figure 19:**
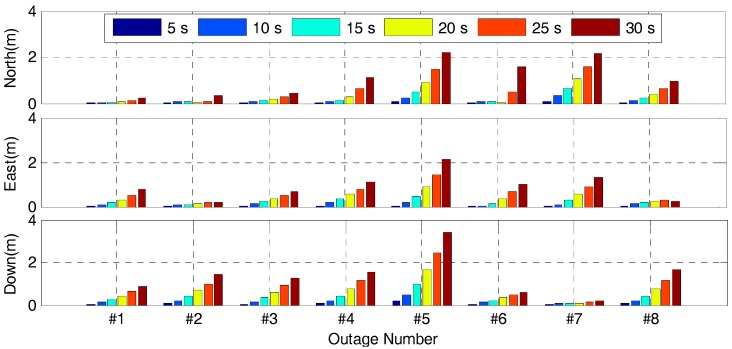
Position drift errors of the tightly-coupled RTK/INS integration during GNSS outage durations of 5, 10, 15, 20, 25 and 30 s using the GPS/BDS/GLONASS observables.

In order to evaluate the INS bridging capabilities of the tightly-coupled algorithm, eight complete GNSS signal outages in different vehicle dynamics were simulated in this test by removing the GNSS update of the Kalman Filter, and each GNSS signal outage has six different outage durations, i.e., 5, 10, 15, 20, 25 and 30 s. Figure 19 shows the position drift errors of the tightly-coupled GPS/BDS/GLONASS/INS integration during different GNSS outage durations. It is clear that the position drift errors for different outage show significant difference, which is mainly due to the different vehicle dynamics and the time-variant IMU errors during different outage period. Obviously, the position drift error will become larger rapidly with the increase of the outage duration.

Table 5 shows the statistics of the position drift error with different GNSS signal outage durations. From this table, we can see that the RMS of position drift errors of the tightly-coupled algorithm in north, east and vertical directions degrade significantly from 4.8, 3.9 and 8.3 cm to 1.352, 1.096 and 1.639 m with the outage duration ranging from 5 s to 30 s, and the maximum position drift errors even increase to 2.196, 2.134 and 3.381 m. During the outage period, the position information is provided by the INS mechanization, and thus the magnitude of the position drift error is mainly dependent on the quality of IMU sensors and outage duration. In this field test, sub-meter position accuracy can be achieved within 15s outage duration.

**Figure 20:**
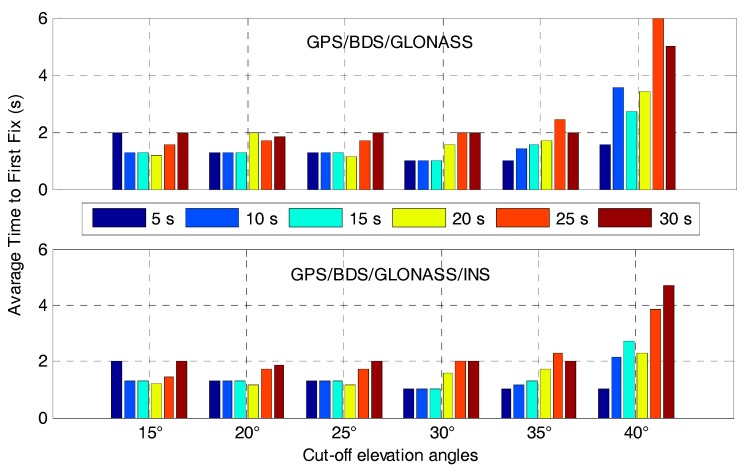
Average time to first fix the ambiguities after GNSS outage durations of 5, 10, 15, 20, 25 and 30 s for the GPS/BDS/GLONASS RTK and the corresponding tightly-coupled RTK/INS integration with elevation cut-off angles of 15, 20, 25, 30, 35 and 40°.

Figure 20 shows the average time to first fix the ambiguities after different GNSS outage durations for the GPS/BDS/GLONASS RTK and the corresponding tightly-coupled RTK/INS integration with elevation cut-off angles of 15, 20, 25, 30, 35 and 40°. It indicates that the recovery of AR within 2 s is possible for short GNSS outage duration when the cut-off elevation angles are between 15° and 35° for the tightly-coupled GPS/BDS/GLONASS/INS integration. Obviously, the performance in terms of average time to first fix shows little difference between GPS/BDS/GLONASS RTK and its corresponding RTK/INS integration when the cut-off elevation angles are between 15° and 35°. This is mainly due to the good geometry strength of multi-GNSS. At 40° elevation cut-off angle, the recovery performance of AR degrades dramatically for GPS/BDS/GLONASS RTK due to the poor satellite geometry strength. By contrast, the AR recovery performance of the tightly-coupled GPS/BDS/GLONASS/INS integration shows improvements when the outage duration is smaller than 20 s because of the strong constraint from INS. If the outage duration continues to increase, this constraint will become weaker and the AR recovery performance degrades further. Generally, the ambiguities could be resolved instantaneously after short outage durations (less than 20 s) for the tightly-coupled GPS/BDS/GLONASS/INS integration when the elevation cut-off angles are between 20° and 35°. This will greatly enhance the system availability in constrained environments.

## 6. Conclusions

In this contribution, we have investigated the benefits of multi-GNSS and MEMS-IMU to enhance the positioning performance of single-frequency RTK. The tightly-coupled multi-GNSS single-frequency RTK/INS integration model as well as the ambiguity resolution with INS aiding is described in detail. A field vehicular test was carried out to validate the ambiguity resolution and positioning performance in different system configurations. According to the analysis and results presented in this research, some conclusions can be drawn as follows:

(1) For the GPS-only system, single-frequency ambiguity resolution is not possible instantaneously in kinematic environment. In contrast, superior single-frequency AR performance can be obtained for multi-GNSS RTK with high empirical success rate (more than 90%) at 30° cut-off elevation angle. In addition, the AR performance and probability of fixing the integer ambiguities are further enhanced after the inclusion of the a priori position constraint from INS, especially in the case of the GPS-only system and higher elevation cut-off angles.
(2) The stability of the position time series and positioning RMS can be greatly improved by combining the multi-GNSS data and MEMS-IMU data. Besides, the position accuracy for multi-GNSS is only slightly degraded even if the elevation cut-off angle is set to 40°, especially for the horizontal components. Hence, continuous and high-accuracy positioning in constrained environment is feasible for single-frequency multi-GNSS users.
(3) INS can provide continuous positioning performance when GNSS positioning is not available, and the magnitude of the position drift error during GNSS outages relies heavily on the quality of the IMU sensor and outage duration. In our case, sub-meter position accuracy can be achieved within 15s outage duration. In terms of the recovery performance of AR, the results show that the average time to first fix the ambiguities after short outage durations (less than 30 s) is within 2 s for the tightly-coupled GPS/BDS/GLONASS/ INS integration when the cut-off elevation angles are between 15° and 35°.

In the future, the rapid deployment of multiple constellations would generate more available observations, which means the AR and positioning performance of the single-frequency RTK can be further improved. Meanwhile, the rapid development of MEMS technology will bring about cheaper but better IMU sensors. Undoubtedly, more potential applications can adopt this technology to provide continuous and high-accuracy positioning.

## Figures and Tables

**Table 1:** Performance specifications of the IMU sensor.

Dimensions (mm)	Bias		Scale Factor Stability		Random Walk	
	Gyro. (°/h)	Acce. (mGal)	Gyro.(ppm)	Acce.(ppm)	Angular (${}^{\circ}/\sqrt{h}$)	Velocity ($m/s/\sqrt{h}$)
168 × 152 × 89	20	50000	1500	4000	0.0667	0.10

**Table 2:** Mean DOPs of GPS/BDS/GLONASS for 15° to 40° cut-off elevation angles.

Cut-Off (°)	15	20	25	30	35	40
PDOP	1.1670	1.3043	1.8347	2.7552	3.4205	4.2781
HDOP	0.5187	0.5579	0.7044	0.9165	1.0135	1.1521
VDOP	1.0433	1.1866	1.6842	2.6033	3.2651	4.1215

**Table 3:** Single-frequency, single-epoch AR empirical success rate for different system configurations with elevation cut-off angles of 15, 20, 25, 30, 35 and 40°. The RMSs of the corresponding number of satellites are shown within parentheses.

System Configuration		Success Rate $P_{SE}$ (%) for Different Cut-Off Elevation Angles (°)					
		15	20	25	30	35	40
RTK	GPS	8.4 (7.8)	4.3 (7.0)	0.4 (5.8)	—	—	—
	GC	86.4 (19.2)	89.5 (18.3)	92.7 (15.6)	92.6 (12.5)	77.7 (10.8)	27.2 (8.5)
	GCR	84.3 (26.4)	88.5 (24.2)	93.2 (19.5)	95.9 (15.4)	90.9 (13.2)	50.3 (10.8)
RTK/INS	GPS	77.7 (7.8)	67.6 (7.0)	6.8 (5.8)	—	—	—
	GC	87.1 (19.2)	90.3 (18.3)	94.1 (15.6)	97.8 (12.5)	99.3 (10.8)	99.7 (8.5)
	GCR	84.5 (26.4)	88.6 (24.2)	94.0 (19.5)	97.7 (15.4)	99.1 (13.2)	99.5 (10.8)

**Table 4:** RMS of the position difference of RTK and RTK/INS for GPS, GPS/BDS, GPS/BDS/GLONASS at 15° cut-off elevation angle.

System Configurations		GPS		GPS/BDS		GPS/BDS/GLONASS	
		RTK	RTK/INS	RTK	RTK/INS	RTK	RTK/INS
RMS (mm)	North	5.8	5.0	3.3	2.2	2.8	1.2
	East	4.0	3.4	2.4	1.7	2.2	1.2
	Down	9.9	8.9	7.3	5.4	6.8	3.6

**Table 5:** Statistics of position drift error of the tightly-coupled RTK/INS integration during GNSS outage durations of 5, 10, 15, 20, 25 and 30 s using the GPS/BDS/GLONASS observables.

Outage Duration (s)		5	10	15	20	25	30
RMS (m)	North	0.048	0.156	0.316	0.535	0.861	1.352
	East	0.039	0.126	0.270	0.481	0.757	1.096
	Down	0.083	0.236	0.473	0.788	1.177	1.639
Max (m)	North	0.101	0.327	0.661	1.087	1.609	2.196
	East	0.054	0.204	0.482	0.886	1.436	2.134
	Down	0.170	0.492	0.986	1.635	2.436	3.381
